# Haydon;—A Psychological Study
*"Autobiography of Benjamin Robert Haydon." Edited by Tom Taylor, 3 vols. 8vo. 1853.


**Published:** 1853-10-01

**Authors:** 


### Art. III.—HAYDON ;—A PSYCHOLOGICAL STUDY.*

Poverty and misery, unceasing struggles for the necessaries of life, and premature death, fall not uncommonly to the possessors of that kind of mental endowment ordinarily termed genius. The usual source of these calamitous circumstances is the want of common sense. Is common sense incompatible then with genius? or are poverty and misery the necessary results of high intellectual labour? Surely, such is not the fact. Let the different fates of Turner and Haydon teach this truth.

In the singular work before us, we have ample materials for a psychological study of genius in its usual course of development and termination. Benjamin Robert Haydon, the historical painter, kept a journal during many successive years of his life, and made it the daily record of his thoughts, aspirations, devotions, designs. Besides this, he wrote his autobiography. This he did for reasons variously stated. "My view," he remarks incidentally in one place, "is to give my readers a notion of my character, temperament, virtues, vices, and infirmities." More formally, in another, he assigns a higher motive—the assertion of a great principle, and the defence of his public life and conduct.

"Every man," he remarks in his introduction, "who has suffered for a principle and would lose his life for its success,—who, in his early days, has been oppressed without ever giving the slightest grounds for oppression, and persecuted to ruin because his oppression was unmerited,—who has incurred the hatred of his enemies exactly in proportion as they became convinced they were wrong,—every man who, like me, has eaten the bitter crust of poverty, and endured the penalties of vice and wickedness where he merited the reward of virtue and industry, should write his own life."

In the same introduction to his "autobiography" from which the above sentence is taken, he assigns another—namely, that his life should point a moral.

"My mistakes, I hope, will be a beacon to the inexperienced, my occasional victories a stimulus to the persevering; and the manner in which I have been elevated from the depths of want and disgrace to the

* "Autobiography of Benjamin Robert Haydon." Edited by Tom Taylor, 3 vols. 8vo. 1853.

heights of fortune and hope, an encouragement to those who believe, as I believe, that bending before the corrections of the Almighty is the only way to save the brain from insanity and the heart from sin."

A weak and delusive faith, indeed, was this of poor Haydon's. No sufferings ever corrected the infirmities of his temper and heart. What, indeed, he calls "the corrections of the Almighty," before which he rarely bent, were nothing more than those results of his own follies which drove him, at last, to suicide. Well—praise and blame are now alike to him; and if, in drawing the true moral from this singular record of a restless life, we take the recorder at less than his own estimation, to him, at least, it is a matter of no consequence.

It will be well, however, to observe by way of introduction, that although Haydon has recorded in his diary much that a man of common sense would have left in oblivion, he has surely omitted to mention much that would have elucidated his own character. It is Epictetus, we think, who remarks that "no one will confess himself a fool or a blockhead, but that, on the contrary, you will hear every one say, I wish my fortune was equal to my mind." This general frailty was a special and prominent characteristic of Haydon's mind. His exaggerated estimate of himself is ludicrous in its simplicity. It would be difficult, however, to say whether he offended most by his quiet assumptions or his passionate dogmatism. Uncontrollable himself, he was utterly intolerant. "Wilkie was content," he remarks, "to do what was wanted to be done in art—I gloried in trying to force people to do what they ought," *i. e.*, to force *his* opinions upon people. No wonder that the sagacious Wordsworth remarked, when he called upon him, in London, in 1824, "Well, Haydon, you found the world too strong." Nothing bore down his self-conceit except his own suicidal hand; he therefore had a "set-to" with Wordsworth. When, in 1826, he exhibited a picture at the Royal Academy, fairly driven to this trivial act of courtesy to the profession by his overwhelming necessities, his professional brethren of the academy reciprocated the courtesy. "The two or three I have spoken to have evidently behaved with great kindness. I see they have a high opinion of me. But no concession; d—— me, if I make any concession." Again, "I think myself the man," he remarks, in 1824, "and I would venture to predict that if the books were open for the public to write the name of the man they think most capable of conducting a great system of art, Haydon would preponderate fifty to one"!

These are some of many illustrations of his inextinguishable vanity and self-will.

Before entering, however, into an analysis of Haydon's mental characteristics, we will trace his career from childhood upwards, introducing, from time to time, such incidental notices of artists' life in general as will illustrate the social and domestic circumstances of that irritable "genus." Haydon was born at Plymouth; his father was a bookseller. He was "well descended and connected" by both his father's and mother's side; his grandmother was Mary Baskerville, a descendant of the great printer. "She was a woman of great energy and violent prejudices," Haydon observes, and probably it was from her that he inherited the same characteristic. She hated the French and she hated the Americans, with much the same ardour as Haydon hated the Academy. It is very probable, therefore, that he was (as he himself believed) "an excessively self-willed, passionate child." From his earliest infancy he displayed an inclination for engravings and for drawing. This was encouraged, when he was afterwards sent to the grammar school, by the Rev. Dr. Bidlake, the master, who always took him and another boy from their studies to attend his caprices in painting; and sometimes taking him to Bickly Vale to sketch and drink tea. He was next sent to a boarding-school, where it was understood that he was not to learn drawing; but his bias to art proved too strong. He spent his pocket-money in caricatures that he might copy them; made the boys into a drawing-class on a half-holiday; sketched a hunt, with burnt sticks, all round the hall; and tried his hand at etching.

Haydon was apprenticed to his father; and with his apprenticeship "began that species of misery," he observes, "I have never been without since—ceaseless opposition. Drawing for amusement was one thing, but studying the art for a living was another. My father's business realized a handsome income; I had nothing to do but pursue his course, and independence was certain." The "ceaseless opposition" thus complained of was, in fact, nothing more than the necessary result of his own ill breeding, folly, and pride.

"I hated day-books, ledgers, bill-books, and cash-books; I hated standing behind the counter, and insulted the customers; I hated the town and the people in it. I saw my father had more talent than the asses he was obliged to bend to; I knew his honourable descent, and I despised the vain fools that patronised him. Once, after a man had offered me less than the legitimate price for a Latin Dictionary, I dashed the book on its shelf, and walked out of the shop." The father's example in this, as in other matters, was lost on the son. "My father," Haydon adds, "restored his customer to good humour, by explaining to him the impropriety of expecting a respectable tradesman to take less than the market-price. The man, convinced, paid the full price and took the book."

This incident ended Haydon's career as an apprentice. His whole mind was now filled with the idea of being a great painter. He got

Albinus' plates, copied them, learnt the origins and insertions of the muscles, and in the course of a fortnight had got them all by heart.

"My anxiety was incessant. My head whirled at the idea of going to London and beginning life for myself. My father had routed me from the shop because I was in the way with my drawings; I had been driven from the sitting-room because the cloth had to be laid; scolded from the landing-place, because the stairs must be swept; driven to my attic, which now became too small; and at last I took refuge in my bed-room."

Arrived in London, he begins work with characteristic energy. For three months he saw nothing but his books, casts, and drawings. His devotion to study, he asserts, was that of a martyr.

"I rose when I woke, at three, four, or five, drew at anatomy until eight, in chalk, from my casts from nine to one, and from half-past one until five—then walked, dined, and to anatomy again from seven to ten and eleven. I was so long without speaking to a human creature, that my gums became painfully sore from the clenched tightness of my teeth. I was resolved to be a great painter, to honour my country, to rescue the art from that stigma of incapacity which was impressed upon it. However visionary such aspirings may seem in a youth of eighteen, I never doubted my capacity to realize them. I had made up my mind what to do. I wanted no guide."

This sentence is the key to his entire career. The same visionary aspirings, the same contempt for a "guide," the same overweening estimate of himself, influenced the whole of his life, was the last sentiment he expressed, and sent him at last to a premature grave.

He got introductions to Northcote and others. Northcote he found a diminutive wizened figure, bald, with spectacles pushed up on his forehead, and clad in an old blue striped dressing gown. In the broadest Devon dialect, he said, when told by the postulant for artistic revolutionary fame, that he meant to be a historical painter—"'Heestoricaul peinter! Why yee'll starve with a bundle of straw under yeer head!' Haydon quoted Michael Angelo in reference to anatomy. 'Michel Angelo,' exclaimed Northcote, 'what's he tu du here? You must peint portraits here!' This roused me, and I said, clinching my mouth, 'But I wont!'—'Wont,' screamed the little man, 'but you *must!* You father isn't a monied man, is he?'—'No, sir; but he has a good income, and will maintain me for three years.' 'Will he? hee'd better make-ee menteen yeeself!'" The advice was lost on the visionary with the fixed idea. Yet he was too glad, eventually, to paint portraits, and—lower degradation—copies of his "Napoleon Musing," &c., by the dozen. He got a letter from Northcote to Smirke, the father of Sir Robert. Smirke laughed at his evident sincerity, and gave Haydon much good advice, which was, of course, lost upon him—"For it was curious," says Haydon, very naively, "the power I had of sifting all advice, and discarding everything which interfered with my own decisions"! Fuseli encouraged him. On calling, he found in Fuseli's gallery or show-room, enough to frighten anybody at twilight.

"Galvanized devils,—malicious witches brewing their incantations,—Satan bridging chaos, and springing upwards like a pyramid of fire,—Lady Macbeth,—Paolo and Francesca,—Falstaff and Mrs. Quickly—humour, pathos, terror, blood, and murder, met me at every look! I expected the floor to give way—I fancied Fuseli himself to be a giant. I heard his footsteps, and saw a little bony hand slide round the edge of the door, followed by a little white-headed, lion-faced man in an old flannel gown, tied round his waist with a piece of rope, and upon his head the bottom of Mrs. Fuseli's work-basket."

He spoke with strong Swiss accent, and swore roundly, a habit he had contracted from associating with Dr. Armstrong. He painted with his left hand, and, being very near-sighted, and too vain to wear glasses, painted much at random. "Sometimes in his blindness he would put a hideous smear of Prussian blue in his flesh, and then, perhaps, discovering his mistake, take a bit of red to deaden it, and then prying close in, turn round to me, and say, 'By Gode! dat's a fine purple! It's very like Corregio, by Gode!' And then all of a sudden he would burst out with a quotation from Homer, Tasso, Dante, Ovid, Virgil, or perhaps the 'Niebelungen,' and thunder round to me with 'Paint that!'" Haydon describes Fuseli as a most grotesque mixture of literature, art, scepticism, indelicacy, profanity, and kindness. Weak minds mistook his will for reason, his indelicacy for breeding, his swearing for manliness, and his infidelity for strength of mind.

Wilkie and Jackson were Haydon's intimate friends and colleagues when a student at the Academy. Wilkie asked him to breakfast one day. "I went to his room rather earlier than the hour named, and to my utter astonishment found Wilkie sitting stark naked on the side of his bed, drawing himself by help of the looking-glass! 'My God, Wilkie,' said I, 'where are we to breakfast?' Without any apology or attention to my important question, he replied, 'It's just copital practice!'" Jackson was perfectly free from envy; he introduced both his friends to his own patron, Lord Mulgrave, who gave Haydon, in consequence, the first commission he received as a historical painter. Jackson was exceedingly indolent, and very fond of idle gossip:—

"Sooner than not gossip," "he would sit down and talk to servants and valets, drink brandy and water with them, and perhaps sing a song. He would stand for hours together with one hand in his trowsers pocket, chatting about Sir Joshua and Vandyke, then tell a story in his Yorkshire way, full of nature and tact, racy and beautiful, and then start off anywhere, to Vauxhall or Covent Garden, 'to study expression and effect.' After some time, Lord Mulgrave thought he had discovered that Jackson was beginning to be idle, which his lordship helped to make him, by sending him constantly to sales. At last his carelessness became so apparent, that Lord Mulgrave, in a passion, cut off his income, and threw him on his own resources. This brought Jackson to his senses. He exerted himself, and he told me that it had saved him. I certainly date his independence of character from that moment, nor was he so weak, but that when he found himself deserted, he dared all sorts of things for an honest subsistence, and found himself happier as his own master."

Although Haydon adds, very characteristically, "I thank God, I never had a patron as he had, and I would have shown the door to any man who had offered such patronage!" yet his delight was in patrons nevertheless. When he came to dine with Sir George Beaumont and Lord Mulgrave, his happiness knew no bounds. Sir George advised him not to send his first picture to the exhibition, and sadly puzzled was the painter whether to obey his patron, or "fly in his face" and risk offending him. He decided to exhibit, however. His description of his feelings and doings is highly characteristic:—

"As the exhibition time approached, I felt all those cursed torturing anxieties that are the bane of this mode of making your name known to your countrymen—a mode the most absurd, unjust, despotic, and ridiculous, that was ever invented by the most malignant in act. I dreamed that the exhibition was open, and that I hurried into the rooms and could not find my picture; that I ran about raving for the porter; and at last found myself in the Academy kitchen, and there, under the table, and covered over with the servants' table-cloth, found my picture, dirty and torn. I became furious, awoke, and found myself sitting bolt upright in my bed; but for some time I could not get rid of the delusion."

If he had been as deep in oneiromancy as in bibliomancy he would (with Rory O'More) have derived consolation and courage from the well known principle in vaticination that dreams always go by contraries, as was certainly the case with his. He had, however, no such lore, so he adds—

"For days I wandered about in hopeless misery; I could not eat nor drink; I lost my relish for everything; I could not sleep, I could not paint; called on one friend after another, affecting gaiety; bored Fuseli, who, being keeper, saw what was daily doing by the committee; until at last, one morning, when, after a timid knock, I opened the door at the usual 'Come in,' Fuseli turned suddenly round with his lion head, the white hair glistening as the light quivered down upon it from the top of his high window and roared out, 'Wale, is it you? for your comfort den, you are hung be Gode, and d—d well, too, though not in chains yet.'—'Where, sir, for God's sake?' 'Ah! dat is a sacrate; but yer are in the great room. Dey were all pleased. Northcote tried to hurt you, but dey would not listene; he said, 'Fye, zure I see Wilkie's hand dere.'—'Come, come,' said Westall, 'dat's too bad even for you!' 'Wilkie's hand,' replied I, 'good heavens, what malice! I would as soon let Wilkie feed me with a pap-spoon as touch a picture of mine. But what petty malignity!'—'Wale, wale,' said Fuseli, 'I told him (Northcote) you are his townsman, hang him wale. When I came back whayre de deyvil do you tink he was hanging you? Be Gode, above de whole lengts and small figures about eight inches. 'Why,' said I, 'you are sending him to haven before his time. Take him down, take him down; dat is shameful!' And so down I was taken, and hung on the right of the entrance-door in the old great room at Somerset House, which for a first picture by a young student, was a very good situation, and obtained me great honour."

It will be well to pause here, and examine into Haydon's prospects. He came to London a stranger and a student; in three years he had obtained a position of great honour in the Exhibition; had secured the society and friendship of men of rank, and of the first artists of the day, and was clearly on the high road to fortune, if common sense had but only directed his steps. This his friends thought, too. He had been to Plymouth to visit his sick father, and there he soon got into full employment at portrait painting, and was paid the handsome sum of fifteen guineas per head. On his return to London he "was welcomed with great affection" by Lord Mulgrave, Sir George Beaumont, Wilkie, and all his other friends. "Strolling out one evening with Fuseli," he observes, "and explaining to him my commissions and prospects, he said, 'I think you may *vainture* now upon a first floor;' so to look after a first floor I went, and found one with every accommodation at 41, Great Marlborough-street."

Thus encouraged, without anxiety in pecuniary matters, with health, and an exquisite sense of enjoyment, Haydon is well justified in describing this period of his life as one of great happiness. His first interview with the Elgin marbles, then but just arrived, and for which he was indebted to Wilkie, is highly characteristic. He felt intuitively the true art-principles which these figures illustrate.

"The first thing I fixed my eyes on, was the wrist of a figure in one of the female groups, in which were visible, though in a feminine form, the radius and ulna. I was astonished, for I had never seen them hinted at in any female wrist in the antique. I darted my eye to the elbow, and saw the outer condyle visibly affecting the shape, as in nature. I saw that the arm was in repose, and the soft parts in relaxation. That combination of nature and idea which I had felt was so much wanting for high art, was here displayed to mid-day conviction.

My heart beat! * * * * * Oh, how I inwardly thanked God that I was prepared to understand all this! how I was rewarded for all the petty harassings I had suffered! Now was I mad for buying Albinus without a penny to pay for it? Now was I mad for lying on the floor for hours together, copying its figures? I felt the future; I foretold that they would prove themselves the finest things on earth; that they would overturn the false beau-ideal, where nature was nothing, and would establish the true beau-ideal, of which nature alone is the basis. I shall never forget the horses' heads—the feet in the metopes! I felt as if a divine truth had blazed inwardly upon my mind, and I knew that they would at last rouse the art of Europe from its slumber in the darkness. I do not say this *now*, when all the world acknowledges it, but I said it then, *when no one would believe me.* I went home in perfect excitement, Wilkie trying to moderate my enthusiasm with his national caution."—Vol. i., p. 85.

There is no reason to doubt so much, at least, of this statement as records Haydon's *feelings.* The course of study he had followed and the natural constitution of his mind combined to develope his intuitive perceptions of what was true in works of art. That he could not combine his own ideas, nor execute them as perfectly as he perceived the truth in others, is no uncommon defect in men of a tumultuous, impulsive nature like his. Lord Mulgrave obtained for him from Lord Elgin the rare privilege of permission to draw from the marbles. This he availed himself of largely, and with his wonted enthusiasm.

"I drew at the marbles, ten, fourteen, and fifteen hours at a time; staying often till twelve at night, holding a candle and my board in one hand, and drawing with the other; and so I should have stayed till morning had not the sleepy porter come yawning in, to tell me it was twelve o'clock, and then often have I gone home, cold, benumbed, and damp, my clothes steaming up as I dried them; and so, spreading my drawings on the floor, and putting a candle on the ground, I have drank my tea at one in the morning with ecstasy, as its warmth trickled through my frame, and looked at my picture, and dwelt on my drawings, and pondered on the change of empires, and thought that I had been contemplating what Socrates looked at, and Plato saw, and then, lifted up with my own high urgings of soul, I have prayed to God to enlighten my mind to discover the principles of those divine things; and then I have had inward assurances of future glory, and almost fancying divine influence in my room, have lingered to my mattress-bed, and soon dozed into a rich balmy slumber. Oh, these were days of luxury, and rapture, and uncontaminated purity of mind! No sickness, no debility, no fatal, fatal weakness of sight. I arose with the sun, and opened my eyes to its light, only to be conscious of my high pursuit; I sprang from my bed, dressed as if possessed, and passed the day, the noon, and the night, in the same dream of abstracted enthusiasm; secluded from the world, regardless of its feelings, *un*impregnable [?] to disease, impassible to contempt, a being of elevated passions, a spirit that—

'Fretted the pigmy body to decay,
And o'erinformed its tenement of clay.' "—Vol. i., p. 88.

In this rhapsody the germs of several points in Haydon's character, to be examined shortly, may be noticed. He was now twenty-two years old, and had risen, without question, very rapidly. Lord Mulgrave had not only got Haydon leave to draw from the Elgin marbles, but had procured him armour from the Tower. He encouraged Haydon in his studies; invited him to dinner with the "familiarity of a friend;" introduced him to the nobility and the most distinguished society, and gave him a position which, with all his errors, he never subsequently lost. Haydon attributes this "victory," as he justly calls it, to his industry, single-mindedness, and integrity. The victory ruined him.

Haydon now began not to relish the society of the middle classes; he thought their manners gross, their breeding hideous. He "talked rather more grandly to the artists." "Sir George would say that he had always said, 'A great historical painter would, at last, arise, and I was he.'" "'We look to *you*, Mr. Haydon,' said a lady of the highest rank once, 'to revive the art,'—then people would whisper, 'he himself has an antique head,' and then they would look, and some would differ." Haydon believed "all this to be gospel truth," and expected "that the Academy would hail with open arms so extraordinary a student." His picture of Dentatus was sent to the Exhibition of 1809; it was hung in the centre of the ante-room—a room, in Lord Mulgrave's opinion, without a window or decent light for any great work. This his patron looked upon as the criticism of the Academy; and, although he paid Haydon 210 guineas for his picture, he became somewhat cool towards his protégé. To Haydon this was a terrible shock. He had been so elevated at the praises of the great, and at the visit of crowds of beauties, putting up their pretty glasses, and lisping admiration of his efforts, that he already believed his fortune to be made. "I walked about my room, looked into the glass, anticipated what the foreign ambassadors would say, studied my French for a good accent, believed that all the sovereigns of Europe would hail an English youth with delight who could paint a heroic picture." But, with the silent criticisms of the Academy, things changed. "People of fashion were ashamed to acknowledge that they had ever seen either the picture or the painter. My painting-room was deserted. I felt like a marked man. How completely the Academicians knew that class whose professions of regard and interest I had credited like a child!"

Haydon was now as readily depressed as he before was elated. He began to think *he was under a curse, and doomed to be so.* He writes, "*My brain was affected; the splendour and refinements of high life dis-*

*gusted me. I felt its hollow glitter, and became sullen, retired, and musingly thoughtful.*" Yet his old friends still stuck to him. Lord Mulgrave gets him a sea-trip in a man-of-war; Wilkie is his companion, and they are treated very handsomely by the people of Devonshire. They visit with Mr. Canning's mother at Bath, and then go for a fortnight to Coleorton, the seat of Sir George Beaumont, where the two passed their time "as delightfully as painters only could." Yet, when Sir George gave him a commission to paint "Macbeth," and displayed divers not unexpected caprices, Haydon has no polite tact, nor even sense of gratitude; and instead of taking Lord Mulgrave's sensible advice, who shook him kindly by the hand, and whispered, "yield," he inflated his vanity, and mounted his high horse. He thus describes his state of mind after parting from Lord Mulgrave—the account of his somnambulism being wofully prophetic of that morbid cerebral condition which finally raised his hand against his own life.

"Up I went to my solitary painting-room, and putting the candle on the ground, dwelt on my picture in its advanced state; mused on the grooms heavy in slumber; the king sleeping in innocence; Macbeth striding in terror; the vast shadow of the listening Lady Macbeth (for at that time I had the shadow alone), till getting inspired as midnight approached, I marched about the room in agitation, and swore I would not yield. Full of the glory of resistance to injustice, I went to bed and fell asleep. In the night I awoke, and found myself standing in my cast-room, where I must have been a long time, half dead with cold, bewildered, and staring at the head of Niobe. The glitter of the moon awoke me. The clock struck three, and I became conscious I had been walking in my sleep. I shivered back to bed, and lay in perfect anxiety till day broke, and then I got up, prayed in distrust, and set my palette. I could not paint; I felt sick; my model came; I kept him standing without speaking, till he remarked my abstraction, and asked me if he should go. I said 'Yes;' and he left me with an expression of surprise, as if he thought me mad, or getting so. All day I stood staring at the picture, longing to proceed, but utterly nerveless, when Wilkie called, and advised me to oblige Sir George by doing a smaller work."—Vol. i., p. 128.

Haydon had evidently given offence to his patron by his arrogance. The difference between them was as to the size of the picture. Haydon had a passion amounting to monomania for "acres" of canvass. Sir George did not desire so large a picture as the painter had commenced and partly completed. Haydon proposed to finish the picture, and if, on seeing it completed, Sir George still objected, he would paint him another on any subject and any scale he pleased. This proposition Sir George accepted, but he was more distant with his disagreeably impulsive *protégé* than heretofore, and Haydon resented this conduct. "At last, wearied out by his apparent want of interest, I talked with great indignation; and talking having no effect, as he still kept from me, I resolved, against all breeding and delicacy, to show the correspondence. This soon got, as I intended, to the ears of the academicians, and then of Sir George." The answer to this escapade was a card of invitation to dinner from his patron, evidently that an opportunity might be afforded of giving the youngster a lesson on the "indelicacy" of showing people's letters. On Haydon it was, of course, lost. He was proud of a quarrel with a man of rank, because "it would help to bring him into notice"! Wilkie told him that flying in the face of the academicians was not the way to get on. Haydon acknowledges that it is all very well to give "a proper rap to a man of fashion; but on whom does the rap strike hardest? On the young student struggling for fame and existence, or the man who has the power of preventing both? Go to work—keep pen and ink out of your painting-room—finish your picture, and let that speak for itself. The longer I live, and the more I see, the more strongly and sincerely can I recommend this conduct to the artist, not only at the beginning of his career, but all through life." The advice is sound, and of general application to all professions. Haydon neglected it to the last!

All this time he had been supported by his father. This help, he remarks, "had continued for six years, and I was anxious to relieve him, but could not, though I might have done so by painting paltry things; but I was iron-minded, and I bent not. I still pressed him, and he still helped me through 1810, though often irregularly." Haydon institutes a comparison between himself and Wilkie, as to their early career; but he hardly does Wilkie justice. Wilkie had no father to help him for six years, nor was he ashamed to labour in the humbler departments of his profession. When Haydon was being supported by his father, or borrowing of Wilkie, and despised to do "the paltry things" which might have made him independent, Wilkie struggled alone, was glad of any employment, and entered into an engagement with an engraver to copy Barry's pictures at the Adelphi. Wilkie got a knocking-down to his vanity just like Haydon, and just like every man who seeks popular applause; and the effect upon his health was great. Some little hitch in court patronage, very insignificant apparently to the unimpassioned observer, overwhelmed him. Haydon says of Wilkie, under these circumstances,—

"I never saw any man suffer so much. From sheer mortification, he sank down to the brink of the grave. Dr. Baillie attended him with great affection. People of fashion crowded to inquire and offer assistance; but he declined any aid lest it might encumber him, and I was so dreadfully embarrassed by the desertion of my father, and my struggles to get through 'Macbeth,' that I could not pay Wilkie the small sum I owed him, much less assist him from my own resources. His situation was distressing. He was too feeble to move. One evening when I called, and was admitted, with a caution as to his danger, I found him lying on the sofa in the attitude of the completest despair I ever witnessed. He had a Prayer-book near him, and his whole air was that of a man who, in his agony, had taken a new and terrible view of human nature. It was weeks before he was quite out of danger."—Vol. i., p. 143.

This incidental remark as to Haydon's inability to repay Wilkie the money he had borrowed is characteristic of Haydon's whole course in money matters. So soon as his father withdrew his help, he fell to borrowing of his friends—of John Hunt, brother to Leigh Hunt, Wilkie, Peter Cleghorn, and others. He prayed to God and borrowed; he ought to have prayed to God, and laboured, but that was not consonant with the pride and vanity which rankled in his heart. Employment in the high walk of art which he had marked out for himself, he had not; nor was he likely to have, when, on any slight or fancied provocation, he quarrelled with his patrons. His popularity with the leading members of his profession, with the men who always will inevitably in the long run lead public opinion as to the merits of a rising artist, may be judged of by this significant entry in his record—"I put my name down this year [1810] for admission to the Academy. Arnold was elected. *I had not a single vote.*" This slight must have been a very grievous wound to his vanity, and was doubtless the secret source of that embittered and unequal warfare which he commenced and long carried on with the Royal Academicians, to his utter ruin. His reckless folly is very manifest from the single circumstance that he was already deeply in debt when he commenced his attack on the Academy in the name of high art. At the end of 1811 (his father had helped up to 1810, and he had got 100 guineas for a prize picture) he writes—"Macbeth being thus concluded, after a long struggle, without assistance from my father, and only by dint of borrowing from my friends, I scrutinized my debts before beginning a new work, and found they were 616*l.* 10*s.*, of which 200*l.* was due to my landlord for rent; and this with no extravagant habits, but solely incurred by the wants of life, and the expenses of the work."

Sir George Beaumont exercised his option of refusal by refusing a picture, for which Haydon demanded "only five hundred guineas," but offered to give him one hundred pounds for the trouble he had had in the commencement of the picture—certainly, under the circumstances, a very liberal donation—which Haydon had the folly and (to his creditors) injustice to refuse. He became furious, and *would* run a muck at the Academy, Sir George Beaumont, and all patrons in general.

From that moment, as he fully acknowledges, the destiny of his life was changed. "He was looked at like a monster, abused like a plague, and avoided like a maniac." He was, in fact, according to his own showing, little better than all three. Yet, to his dying day, he never saw, or, if he saw, never frankly acknowledged, that "not a single vote" at the Academy in 1810, was the real source of all that warfare and all those feelings of hostility and revenge which made his life miserable. We have seen that he was hereditarily a good hater; that he was thoroughly obstinate, irremediably egotistical—*hinc illæ lachrymæ;* so that while he was befooling himself with the notion that he was the apostle and martyr of high art, the public and his enemies only laughed at the disappointed egotist butting against a rock. Nevertheless, by tacking the assertion of a great principle to his private revenge, as many a revengeful clever man has done before him, he did good service to art, although he perished in the conflict.

Haydon's career has from henceforth to be threaded through a long series of pecuniary embarrassments, enlivened occasionally, it is true, by flashes of wonderful success, but only at long intervals. He never thought, however, of freeing himself from these his early debts by an effort of steady industry, but immediately commenced another grand historical picture, "The Judgment of Solomon." At this time he had not for weeks a shilling that he had not borrowed, or got from selling "book after book, clothes, everything;" and being in urgent distress, his thoughts turned to Wilkie.

"First, however, I went to a friend, and said, 'What is to be done?'—'That I can't tell you,' said he, with a cold withdrawing air. I left him in pain, and walked quietly to Wilkie. I told him I wanted the common necessaries of life. He looked at me with horror. I said, 'Will you advance me 10*l.* in addition to the 24*l.* I owe you? He shook, got nervous, was oppressed by my presence, looked cold, heartless, distant, and fearful I would stay long. He stammered out he could not spare more. I urged on him that he risked all by not helping me now. He persisted he could not. He kept saying, 'I told you so, I told you so.' "—Vol. i., p. 179.

There is no wonder that his friends shunned him. A man rubbing in a picture which is to occupy two years, without a shilling, and deeply in debt, "entangled with an infernal woman," or plainly, keeping a mistress, and yet providing for the future in no other way than by sponging on his more really industrious and frugal friends, was not an acceptable acquaintance. He succeeded, unfortunately for himself, but too well. His landlord, already a creditor for 200*l.*, deliberately gave him credit for two years more; and the proprietor of the eating-house, where he always dined, was equally generous. The account of this latter affair is very characteristic:—

"I went to the house where I had always dined, intending to dine without paying that day. I thought the servants did not offer me the same attention. I thought I perceived the company examine me—I thought the meat was worse. My heart sank as I said, falteringly, 'I will pay to-morrow.' The girl smiled, and seemed interested. As I was escaping with a sort of lurking horror, she said, 'Mr. Haydon, Mr. Haydon, my master wishes to see you.' 'My God,' thought I, 'it is to tell me he can't trust!' In I walked like a culprit. 'Sir, I beg your pardon, but I see by the papers you have been ill-used. I hope you wont be angry—I mean no offence; but—you wont be offended—I just wish to say, as you have dined here many years, and always paid, if it would be a convenience during your present work, to dine here till it is done—you know—so that you may not be obliged to spend your money here, when you may want it—I was going to say you need be under no apprehension—hem! for a dinner.' My heart really filled. I told him I would take his offer. The good man's forehead was perspiring, and he seemed quite relieved. From that hour the servants (who were pretty girls), eyed me with a lustrous regret, and redoubled their attention. The honest wife said, if I was ever ill she would send me broth or any such little luxury; and the children used to cling round my knees, and asked me to draw a face."—Vol. i., p. 180.

It is to be hoped he drew all their faces.

Haydon worked day after day at his "Solomon" in a small room, in which he remained for eighteen hours out of the twenty-four. His breakfast cloth, blankets, and sheets, took their turn on a wooden lay-figure: "yet nothing,' he remarks, "could equa my happiness in painting. Oh! I have suffered much, there can be no doubt, but I have enjoyed more; and if I had suffered twice as much as I have enjoyed, my enjoyments are so intense that they amply compensate me." These delights were only on the large canvas; he was miserable when painting small subjects. All this while he had not a sixpence but what he borrowed. There is nothing, indeed, in this autobiography has struck us more than the graceful liberality with which the professional class, not less than the wealthy, supplied the wants of this enthusiastic, impulsive, but vain and weak man. His earnestness covered a multitude of faults—even the greatest of all—the meanness with which he occasionally attempted to escape from his difficulties. The gifts he received from private individuals must have amounted to a large sum in the aggregate. The Hunts nobly assisted him, "at the cost of great personal deprivation; Hilton, just saved from ruin by the sale of his "Mary Anointing the Feet of Christ," offered him a large sum out of the proceeds; West, the President of the Academy, spared a gift from his own limited resources, to keep, as he said, "the wolfe" from his door. Sir Charles Bell, too, did his best; and Wordsworth wrote him sonnets. Although he sold "Solomon" for six hundred guineas, he got no commission, and was as deeply in debt as ever. With the avowed knowledge that "the first moral duty is honestly to provide oneself with bread and cheese," he did not so provide, but neglected the duty that he might "persevere in a great plan which is for the public benefit;" so instead of denying himself, and painting a portrait or a small picture now and then, he dashed at a large canvas to enjoy the excitement, and live on his friends—and the "money-lenders." His lax principles as to borrowing, and his notions of the dealers in money, are eminently characteristic. He goes to one of them and gets 100*l.* in hard cash, acknowledging that he did not know how he was to pay the debt at the time he contracted it, yet because the dealer, with instinctive sagacity, required an extra premium to cover the risk, he was a "reptile," had a "mean, skinny, malicious face," &c. "Too proud," he remarks, "to do small modest things, that I might obtain fair means of existence as I proceeded with my great work, I thought it no degradation to borrow, to risk the insult of refusal, and be bated down like the meanest dealer." The bills he thus accepted became due, and then he had to borrow again wherever he could to take them up. Sir George Beaumont, amongst others, responded liberally to his applications, and at the same time most kindly urged him to "paint fancy heads and smaller works, not less as a means of existence, but as a relief from severer studies." But this was an effort he could not make, and he excused himself on the ground that he had claims for government support, and could not thus yield the question of his rights! Harman advanced 300*l.* Coutts sent him 400*l.*, with a kind letter, saying that he had assisted several in Haydon's line, in the course of a long life, but their prospects were disappointed, and his money lost. He had hardly got this money passed to his account when he gave a dinner to Wordsworth, Keates, Charles Lamb, &c.—paid for, of course, out of the money which was to be devoted to his "necessities."

"On December 28th, the immortal dinner came off in my painting-room, with Jerusalem towering up behind us as a background. Wordsworth was in fine cue, and we had a glorious set-to—Homer, Shakespeare, Milton, and Virgil. Lamb got exceedingly merry and exquisitely witty; and his fun in the midst of Wordsworth's solemn intonations of oratory, was like the sarcasms and wit of the fool in the intervals of Lear's passion. Lamb soon got delightfully merry. He made a speech, and voted me absent, and made them drink my health. 'Now,' said Lamb, 'you old lake poet, you rascally poet, why do you call Voltaire dull?' We all defended Wordsworth, and affirmed there was a state of mind when Voltaire would be dull. 'Well,' said Lamb, 'here's Voltaire—the Messiah of the French nation, and a very proper one too.' He then, in a strain of humour beyond description, abused me for putting Newton's head into my picture. 'A fellow,' said he,

'who believed nothing unless it was as clear as the three sides of a triangle.' And then he and Keats agreed he had destroyed all the poetry of the rainbow, by reducing it to the prismatic colours. It was impossible to resist him, and we all drank 'Newton's health, and confusion to mathematics.' It was delightful to see the good humour of Wordsworth in giving into all our frolics without affectation, and laughing as heartily as the best of us. By this time other friends joined, amongst them poor Ritchie, who was going to penetrate by Fezzan to Timbuctoo. I introduced him to all as a gentleman going to Africa. Lamb seemed to take no notice; but all of a sudden he roared out, 'Which is the gentleman we are going to lose?' We then drank the victim's health, in which Ritchie joined."—Vol. i., p. 355.

Poor Lamb! if the future could have been represented at that party, a death's head grinning behind all, except Wordsworth, would have very appropriately represented it.

His picture of "Christ's Entry into Jerusalem" was as successful as a picture could be, and added largely to his reputation. His net receipts in London from the exhibition of this picture amounted to 1298*l.*; yet he was still in debt. Nobody bought it: it was too large, in fact, for any ordinary building. Sir George Phillips had given him a commission for five hundred guineas; he painted the picture too large for the house; and yet the next picture he commenced with (Lazarus) he determined to make his "grandest and largest work"! The canvas at which he "dashed" was 19 by 15 feet, and he acknowledged that if his room had been large enough, he would have had it more than ten times the size. Such was the wondrous folly of this wild genius.

It was at this time (1821) that we find the first allusion to suicide. The sentiments he expresses are not dissimilar from those he avowed just before the idea passed into action. It is interesting, psychologically, to trace its progress.

"I am inclined to imagine that much of the *pain and anxiety of mind* I have suffered for the last few days arose from nothing more or less than *indigestion. My stomach was heated, and affected my brain.* Suppose in that humour I had shot myself? *Would a Superior Being have destroyed my soul, because, my brain being irritated by indigestion, I had in a state of perturbation put an end to a painful existence? Surely not!*"—Vol. ii., p. 15.

It is curious to observe how frequently Haydon recurs to the thoughts of suicide after this questioning fashion. "I am sorry to say," he writes soon after this, "that I am not so convinced of the wickedness of suicide as I am of its folly."

His editor attributes this state of mind to the conjoint influence of his pecuniary anxieties, and his longing to marry the lady who, afterwards, was the suffering and courageous partner of his terrible vicissitudes. His cerebral powers at this time were evidently exhausted; nature stepped in to the rescue, and he idled about for some weeks, doing nothing. The rest thus instinctively secured had its effect, and he fell to work with renewed energy. Twenty-five years later the same feebleness came on, but, alas! he was older, his begging letters were unproductive, had a family, and his overwhelming necessities urged him on to labour, although staring like an idiot at his picture for hours together. Haydon was married in 1821, and from that year to his death he had few respites from pecuniary difficulties. He suspected any proffered kindness, pestered all classes about his misfortunes, and quarrelled with the best of his friends, if they turned a deaf ear to his requirements. One or two instances may be mentioned to illustrate his want of common propriety in this respect. Wilkie is the sufferer in the first we shall notice; and the quarrel was about an arrest.

"He had been my old friend. He had dined with me the night before. He had drank success to my marriage. We parted mutually friendly. The next morning I was arrested by a printer, to whom I had paid 120*l.* that year, for the balance of 60*l.* It was the second time in my life. The bailiff said, 'Have you no friend, sir?' 'Certainly,' said I, and at once drove to Wilkie's. 'Where ought I to have driven? Where ought I to have thought of?' 'I thought it would come to this,' said Wilkie, and after a great deal of very bad behaviour he became my bail. When roused, I am like a furious bard of ancient days. I poured forth such a dreadful torrent of sarcasm and truth that I shook him to death. Wilkie told me to-day it sunk deep into his mind, and never left him for months."—Vol. ii., p. 211.

The other instance is that of Haydon's conduct to his landlord, Newton. Haydon's editor says, that Haydon was in the constant receipt of singular kindnesses from Newton, "who forebore to press him for heavy arrears of rent," . . . "who was always ready to advance him money in his worst emergencies." Yet upon some little hesitation on the landlord's part to let Haydon have his own way—"the greatest of all blessings," as he in one place emphatically declares—Haydon wrote him a letter, part of which we subjoin. The first portion refers to some trivial "gossip and scandal."

"And this is the way to excuse your own abominable cruelty, in doing your best to add to the weight of degradation and misery I have suffered by insinuating to my wife these abominable lies. . . . . Don't talk to me of your affection. Pooh! To let a friend come out of prison after ten weeks' locking up—degraded in character, calumniated and tortured in mind—to let him come to what had hitherto been the solace of all his distresses (his painting room) stripped of all that rendered it delightful, and stripped, too, under the smiling pretences of friendship, and under the most solemn assurances that everything would be returned, and then, on the very morning I came home, when one would have thought all beastly feelings of interest would have been buried in the pleasure of welcoming me back, at such a moment to break your word, and to add to my forlorn wretchedness by refusing to keep it, is a disgrace to your heart and understanding, and will be even after you are dead, as well as while you are living———." Vol. iii., p. 53.

Haydon's editor very justly remarks of this letter (and there is much more in the same style as the extracts above given) that it could not have come from a man "with the views usually prevalent about money obligations." That Haydon's notions of these were very lax is evident from various circumstances. He induced his pupils, for example, to accept bills for his accommodation, and he paid his debts by borrowing more —after the most approved fashion of the borrowing tribe. This he "called doing his duty." On February 3rd, 1843, he has an entry to this effect:—

"In one hour and a half I had 10*l.* to pay upon my honour, and only 2*l.* 15*s.* in my pocket. I drove away to Newton, paid him 2*l.* 15*s.* and borrowed 10*l.* I then drove away to my friend, and paid him the 10*l.* and borrowed 5*l.* more, but felt relieved I had not broke my honour [!] Then home, took out all my proofs, called on my subscribers, and saw them left. Thus I have done my duty to everybody to-day; and what is life but a struggle of duty to your God, your country, and your species, day and night, till death?"—*Ibid.*, p. 223.

These peculiar notions of "honour" and "duty" were (Haydon thought) quite consistent with a devout frame of mind. He had, indeed, a peculiar religious turn of feeling, too, for his journals are constantly interlarded with prayers and aspirations—some of them very characteristic of the man—most of them what his editor tersely characterizes as "begging-letters despatched to the Almighty." He had communion with God, but it was mainly about his necessities and his vain notions of high art, himself its self-appointed representative and apostle. "*The moment I touch a great canvas, I think I see my Creator smiling on all my efforts. The moment I do mean things for a subsistence I feel as if He had turned his back, and what's more, I believe it!*" He therefore did not hesitate to deceive his employers when painting portraits to discharge his debts. "Finished one cursed portrait—have only one more to touch, and then I shall be free. I have an exquisite gratification in painting portraits wretchedly. I love to see the sitters as if they thought, can this be Haydon's—the great Haydon's painting? I chuckle. I am rascal enough to take their money, and chuckle more!" When an artist agrees to paint a portrait (or any other work) for a fixed sum, he engages to bring all his genius and skill to bear upon the work, unless he has distinct charges for different styles of "finish." To do otherwise is dishonest. Haydon's estimate of his own conduct

—witty as he thought it—is therefore true enough. In his religious system, however, this kind of conscientiousness had no place. His deity was in fact his own morbidly proud spirit and impulsive desire for enjoyment—this was the "God" which he felt turned his back on him, when labouring at his art, in the humble but honest character of a bread-winner. He could do an undignified thing if it fell in with his ruling passion. To be invited to lecture to "soften costs" of law proceedings squared with his vanity—to paint small subjects or portraits was against the grain. The lecture to "soften costs," is an amusing incident.

"January 2nd [1837.]—Spent yesterday at Hamilton's. Read a lecture to-night to some society at 16, Tower-street—to my infinite amusement at the intense attention paid to me by a set of dirty-faced journeymen, and two servant girls. I had promised a young attorney to do so, and kept my word. It is extraordinary to think of. When I really made a good hit, I saw all the room nodding. It was an eating-house till six—when the master (a member) cleared out for a lecture, and lent it for nothing. The company filled the boxes, and I was placed at the head on two or three boards.

"I was shown up into a library, where was a likeness of Tom Paine. I saw I was in a scrape. If that had been the room, I would have insisted that the fiend should be taken down, or I would have left the room. This comes of promising young attornies, to soften costs, without inquiring character."—*Ibid.*, p. 58.

Haydon, in the eating-house, indeed, was something like Mantalini at the mangle.

To give an idea of the prayers with which his autobiography and journals are interlarded, it will be necessary to extract one or two. The following is of the date February 28th, 1823; it is for deliverance from a "villain," or creditor. He was arrested a few weeks afterwards, so that the prayer, like the petitions to Jove sent up by the heroes of Homer and Virgil, was lost in air.

"O God, Thou who has brought me to the point, bring me through that point. Grant, during the exhibition [of "Lazarus"] nothing may happen to dull its success, but that it may go on in one continual stream of triumphant success, to the last instant. O God, thou knowest I am in the clutches of a villain; grant me the power entirely to get out of them, for Jesus Christ's sake. Amen. And subdue the evil disposition of that villain, so that I may extricate myself from his power, without getting further into it. Grant this for Jesus Christ's sake. Amen with all my soul."—Vol. ii., p. 47.

The next is a prayer for a grand and triumphant blessing on a picture.

"Oh, Almighty God! it is now thirty years since I commenced my picture of Solomon; though deserted by the world, my family, father, friends, Thou knowest well that I trusted in Thee; that Thou didst whisper me to endure as seeing One who is invisible: Thou knowest I never doubted, though without money, though in debt, though oppressed.

"I prayed for Thy blessing on my commencing labours, Thou carriedst me through to victory, and triumph, and exultation.

"I am this moment going to begin a grand work of 'Alexander and the Lion;' bless its commencement, progression, and conclusion, as Thou blessedst 'Solomon.' Grant, in spite of whatever obstruction, I may bring it to a grand and triumphant conclusion. I have my intellect, my eyes, my health, my head, my strength. Confirm my piety, and grant, O Lord, that this work may advance the feeling of my great country for high and moral art, and that I may not be taken till art be on a firm foundation, never to recede, and that I may realize all my imagination hoped in my early youth, for Jesus Christ's sake. Amen."—Vol. iii., p. 188.

The prayer did not, however, speed the work.

"—— Sua cuique exorsa laborem

Fortunamque ferent. Rex Jupiter omnibus idem."

The picture is finished, sent for exhibition, and rejected. "'Alexander' they have not hung at the gallery. I fear some prejudice. They took 'Napoleon' and 'Saragossa,' which are old pictures, but declined hanging 'Alexander.'"!

Haydon's practice of *bibliomancy* arose out of the same erroneous views of the divine relations as the records of prayers. He would open his Bible in the dark, and take the passage which first struck his eye as a species of revelation. There are several instances of this kind recorded, and it would appear from the following entries that something of the kind was customary with him:—

"January 1st.—I arose at daylight, dressed, and going into the parlour as usual, opened the Bible almost in the dark, turned it on its face, and waited for light. I then, getting impatient, lighted a candle, and read, 'Let thy mercy, O Lord, be upon us, according as we hope in Thee.' And now to set my palette, and to work. Half-past eight."—*Ibid.*, p. 93.

"25th.—In the middle of the night I awoke rather depressed from the multiplicity of anxieties. I put my hand on the Testament I always sleep with, and opened a passage in the dark, folded down the leaf, and at daylight found this blessed consolation, 'And our hope of you is stedfast, knowing that as ye are partakers of the *sufferings*, so shall ye be also of the *consolation*.'"—*Ibid.*, p. 219.

This differed in *spirit* in no respect from the ancient method of divination by auguries; indeed he evidently believed in omens. Having an exhibition of two pictures in the Egyptian Hall, he enters:—"4th. * * Omens of failure in this exhibition. 1st. The cab-horse slipped on the wood, and tumbled. 2nd. I let all the letters tumble for the private day, and to-day, in trying to put up Wordsworth, he tumbled, knocked down Lord Althorp, broke the frame, and played the devil. After this, what success can come? Do I believe this, or don't I? Half inclined * * * 8th. Fine. Receipts worse. Is it not funny my writing down these omens? They have turned out so correctly forerunners of evil."

> "Multa modis simulacra videt volitantia miris,
> Et varias audit voces, fruiturque Deorum
> Colloquio."

Haydon's notions as to *suicide* would lead us to the conclusion, if we could believe that his intellect was in a sound state when he gave utterance to his sentiments, that he *had really no sound religious principles.* We have already noted the first traces of that morbid cerebral condition which at last led him to self-destruction. Several times there are entries in his journals which indicate the access from time to time of that same morbid state. "Like Johnson in hypochondria," he remarks, "*there I sit staring, idle, gaping, with not one idea!*" This condition of the brain was doubtless of an *asthenic* character, and was the consequence of over-work and over-excitement. Three of his children, probably begotten when he was in a state of cerebral irritation, died of *hydrencephalus;* and considering the wear and tear recorded, it is not surprising that his own brain at last gave way. In 1841, Wilkie died, and we find about the time (beginning of summer) that Haydon's melancholy usually affected him most, several entries showing the enfeebled condition of his mind, and how much the death of his friend touched his feelings. In May he dreamt of him, and that he addressed remonstrances to him. Wilkie's presence even haunted his waking hours. "I hear his voice fifty times a-day," he says. In June and July this state of feeling continues; and with it a hypochondriacal state of mind, in which the thoughts evidently reverted to suicide. On July 9th he indulges in the following sophistical reasoning:—"*It may be laid down that self-destruction is the physical mode of relieving a diseased brain, because the first impression on a brain diseased, or diseased for a time, is the necessity for this horrid crime. There is no doubt of it.*" Again, in 1845, he remarks—"Good heavens! Gurwood has cut his throat * * * Where is the responsibility of a man with a mind so easily affected by body? Romilly! Castlereagh! Gurwood!"

In the early part of 1846 (the year of his death), we find extracts from his journal, premonitory of the catastrophe about to take place.

"February 5th.—*O. O. O. I sat all day and looked into the fire.* I must get up my third canvas, or I shall go cracked * * *" "*I stared like a baby, and felt like one.*" Perhaps this paralysis was nature's repose. A trip to Edinburgh set him up again; but his anxieties, disappointments, and difficulties came upon him again with overwhelming force. On May 1st, again we have an unmistakeable indication of the disordered state of poor Haydon's brain.

"I set my palette with a disgust, and yet under an irresistible impulse. * * * *I felt my heart sink, my brain confused*, as I foresaw ruin, misery, and a prison! It was hoisting the standard. This is temper. I went on with my palette in a giddy fidget. I brought it out, and, looking at my great work, rejoiced inwardly at the coming background. But my brain, harassed and confused, fell into a deep slumber, from which I did not awake for an hour. I awoke cold, the fire out; but I flew at my picture, and, dashing about like an inspired devil, by three had arranged and put in the alteration."

There was no rest for him, this time. On June 11th, he says—"How I shall manage to get seven hours' peace for work, and yet satisfy my creditors, heaven only knows." On June 16th, he sits from two till five staring at his picture like an idiot"—(the old symptom)—his brain "pressed down with anxiety, and anxious looks of my dear Mary and children." On the 18th, "Good-hearted Newton"—(his landlord)—"don't put in an execution." Who replied, "Nothing of the sort." On the 21st, "Slept horribly. Prayed in sorrow, and got up in agitation." On the 22nd, "God forgive me, Amen. Finis of R. B. Haydon. 'Stretch me no longer on this rough world.'—*Lear*. End of twenty-sixth volume."

We subjoin his editor's account of the catastrophe:—

"This closing entry was made between half-past ten and a quarter to eleven o'clock, on the morning of Monday, the 22nd of June. Before eleven the hand that wrote it was stiff and cold in self-inflicted death. On the morning of that Monday Haydon rose early and went out, returning, apparently fatigued, at nine. He then wrote. At ten he entered his painting-room, and soon after saw his wife, then dressing to visit a friend at Brixton, by her husband's special desire. He embraced her fervently, and returned to his painting-room. About a quarter to eleven his wife and daughter heard the report of fire-arms; but took little notice of it, as they supposed it proceeded from the troops then exercising in the park. Mrs. Haydon went out. About an hour after, Miss Haydon entered the painting room, and found her father stretched out dead, before the easel on which stood his unfinished picture of 'Alfred and the First British Jury'—his white hairs dabbled in blood, a half-open razor, smeared with blood, at his side; near it, a small pistol recently discharged; in his throat a frightful gash, and a bullet-wound in his skull. A portrait of his wife stood on a smaller easel facing his large picture. On a table near was his Diary, open at the page of that last entry, his watch, a prayer-book, open at the gospel for the sixth Sunday after the Epiphany, letters addressed to his wife and children, and this paper, headed, 'Last thoughts of B. R. Haydon, half-past ten.'"—Vol. iii., p. 319.

His last thoughts were, in fact, an acknowledgment that the deed he was about to do was evil; an apology for his conduct to the effect that if he had been encouraged "nothing but good would have come from him;" and a prayer that God would "forgive the evil for the sake of the good." From his *death* we will draw no other moral than this, that his principles of action were the moving cause of his failures and his fate.

From Haydon's *life* we must, however, deduce a more extended judgment: its records were kept by him for that express purpose; and his death was too costly an experience to be neglected. As to the moral, intellectual, and instinctive characteristics of the man, we may affirm that he had naturally a powerful appetency. The love of pleasure, or rather of pleasurable excitement, that arose out of this, did not take a vicious direction, because the action of the representative intuitions was in him so predominant over that of all others as to largely exclude the influence of the merely vicious desires. Every page of these volumes indicates that to represent his ideas on canvas was a passion. We have already adduced facts sufficient to show the early workings of this bias; a later and highly illustrative example may be adduced to indicate its full operation on the whole man.

"April 18th, [1845].—Worked with such intense abstraction and delight for eight hours, with five minutes only for lunch, that, though living in the noisiest quarter of all London, I never remember hearing, all day, a single cart, carriage, knock, cry, bark, of man, woman, dog, or child.

"I washed, dressed, and walked, and when I came out into the sunshine and the road, said to myself, 'Why, what is all this driving about?' though it has always been so for the last twenty-two years—so perfectly delightfully, and intensely, had I been abstracted. If that be not happiness, what is?

"My notion of supreme happiness is a splendid lot of drapery splendidly set on your lay figure; a large picture which shuts you in, just close enough to leave room to paint it; a delicious light, and conscious power of imitation. You go on like a god, spreading your half tint, touching-in your lights and your darks. There is hardly an effort —no anxiety, no fear, no apprehension."—Vol. iii., p. 274.

That this was not an accidental condition is manifest from other entries. Twenty-eight years previously to this entry (1817), we find a similar record. He was then painting his picture of "Christ's Entry into Jerusalem," and he remarks, "I drove at my picture once again. The subject grew on my imagination. I used to retire to rest positively weighed down by the scene—the tumultuous roaring of the crowd—the moving deity in the midst—filled my soul to positive aching. I daily arose and worked with an intensity hardly to be credited."—(Vol. i., p. 345.) Many similar records might be quoted.

No doubt these are correct descriptions of the delight arising from the instinctive operation (for such it is) of any intuitive ideas on the intellectual consciousness. Newton had no less pleasure in his mathematical abstractions, Beethoven and Mozart in their musical, Coleridge in his philosophical. This *reflex action* of the brain is not, however, its highest attainment, *as a whole*, for it leaves the man at the mercy of all modes of impulsive ideas. It is developed at the expense of a disciplined will, and of that combined action of all the faculties which, working in harmony with intuitions as perfect from every source as from *the one*, makes manifest the perfect man. To these impulses poor Haydon was subject; this undisciplined will was his constant bane.

The impulsive bias of Haydon's mind was, of course, shown principally in those characteristics which were most prominent,—namely, his love of representative art, his self-esteem, his pride, his firmness, amounting to obstinacy, his combativeness—either singly or altogether. The impulse is, sometimes, a driving general fury, and he works thirty out of the eight and forty hours.

"With visions of ancient heroes, pictures of Christ, principles of ancient art, humorous subjects, deductions, sarcasms against the Academy, piercing remembrance of my dear children, all crowding upon me, I paint, write, conceive, and fall asleep, start up refreshed, eat my lunch with the fierceness of Polyphemus, return to my room, go on till near dinner, walk, dine, read the paper, return to my study, complete what I have been doing, or muse till dusk, then to bed, lamenting my mortality at being fatigued. *I never rest, I talk all night in my sleep, start up; I scarce know whether I did not even relish ruin as a source of increased activity.* 'Rest, rest, perturbed spirit!'"—Vol. iii., p. 83.

So it was with other matters; he "dashes at his canvas like an inspired devil," he "drives" at it, he "flies" at it. This constantly. He continually "races" into the city for money; and if his earliest, his kindest, his very judicious friend hesitates a moment to minister to his wildly incurred necessities, he "pours forth a dreadful torrent of sarcasm" and shakes the unfortunate man "to death." This activity he called "virtuous industry;" it was more nearly allied to the delirious activity of insanity; it had its source in self-gratification—not self-denial; painful labour (as we have seen) he abhorred.

Haydon had his quiet moments, nevertheless; and in these he saw and felt how ruinously injudicious was his conduct to the academicians, to his patrons, to his friends. One demon was laid, however, only that another might re-appear. Thoughts of the concessions that are always necessary to peace after war, excited his pride and his obstinacy. He remembered the solemn declarations of unalterable hostility he had made in the heat of combat, and with what unqualified condemnation he had uttered his judgment. His vanity whispered, "What will Mrs. Grundy say to concessions?" his obstinacy responded to the whisper. To seek relief from these conflicting emotions, he would "rush" to the source of his intoxication, and "dash" at some large canvas and grand subject, hoping thereby, in his vanity, to place his reputation on such a dizzy height as to compel academicians, patrons, public, to fall down simultaneously and worship him. And this was repeated often. It was when in the midst of these struggles that he usually put up his fervent prayers for victory—with a "God grant it! Thou knowest I have never given in!"

Perhaps there is no branch of art which requires so harmonious and complete a development of all the intellectual powers as that which Haydon chose. Systematic culture, and disciplined faculties, with free scope of action, are essential to success. Haydon, like all self-taught men, thought energy and perseverance all-sufficient, and was neither systematic in his studies, nor amenable to discipline in the exercise of his powers. The consequence was, spasmodic efforts to attain excellence ending in results, the great characteristic of which was a want of taste. It is not probable, indeed, that "a boisterous and combative martyr," as Haydon's editor designates him, could ever attain to that intuitive perception of the *fitness of things* which is the foundation of true taste; it is equally improbable that the efforts made under the violently conflicting emotions which impelled him to labour could ever end in manual excellence—in that power which the perfect artist ought to possess of transferring to canvas and representing thereon the intuitions of the representative faculty. The material organ—the cerebrum—was too tried and shaken by his undisciplined conduct for such excellence to be attainable by him, even if that organ had been less inharmoniously developed than it evidently was. It was physically incompetent to the task; it tottered from time to time (as we have seen) when unusually weighted, and at last, at the time that imperious necessities rendered the needful repose no longer attainable, it failed under the pressure. This must always be the inevitable termination of men organized and acting like Haydon. *Suicidal* monomania may not, indeed, be the specific form of the cerebral disease, but cerebral disease in some form, surely ends their career.

In his moral sentiments, also, Haydon was inharmoniously constituted. Those in relation to the instincts of propagation operated impulsively like all the rest, but the impulse given by these is naturally for beneficent ends: hence he was a kind father and husband. In his friendships, no instinct guided the impulse, except his love of pleasure and self-gratification: hence his treatment of Wilkie, of Sir George Beaumont, of his landlord, Newton, and others. In his letters to men of rank, " an unbecoming familiarity alternated with gross servility;" so in his public appeals, there was a "turgid and undisguised expression of his own exaggerated estimate of his works," not so becoming, by any means, to the scholar and gentleman, as to the unblushing empiric. To the same mental defects may be ascribed that constant reliance upon the government or his friends for support—a reliance which, to the experienced eye, is a sure mark of mental weakness. It is never so with the true hero—the man *totus teres atque rotundus;* he is *self-*reliant, not in word but in deed; and no conventionalities are to him mean, unless they be base. Such an one would, if an artist, see no degradation, certainly, in painting small subjects; nor, if he had a great object in view (as Haydon imagined he had), would matters of mere personal feeling stand in the way of his attaining it. Above all things, the true man would never have weakened the force of his moral influence by degrading appeals to the mercy of his creditors, or the pity of his friends.

There was the same defect in Haydon's representative faculty as in the others. Mr. G. F. Watts remarks, on his pictures (in his estimate of Haydon as an artist), that "their want of beauty repels, and their want of modesty exasperates. Perhaps their principal characteristic is want of delicacy of perception and refinement of execution." Entries in his journals show that the prevailing tone of his mind was not in harmony with ideas of this kind. He strained after grandeur—the tumultuous, appalling, grotesque: and painting often in a state of mind not unlike that of intoxication, there is no wonder that "he daubed and scrawled his brush about" without that delicate intuition of beauty which must be felt, and realized on canvas, to constitute the true genius in art. When his mind is unduly excited or unstrung, we see how constantly the more sombre and repelling ideas arise. He would draw "a gigantic limb" on the wall of Westminster Abbey, "dash in" a Christ nine feet high; wonder what the fish thought of Wilkie, "with their large glassy eyes, in the gurgling deep;" and paint with his brain seething with dim ideas of sombre grandeur, or of the horrible; but rarely, except in his most joyous moods, was his mind the dwelling of the really beautiful. Such as he was through life, such he was at death. His prayer-book was open, as we have seen, at the gospel for the sixth Sunday after Epiphany. Doubtless that sublime and noble passage from Holy Writ was in consonance with the suicidal emotions then unhappily present to his mind, and charmed him with its gloomy grandeur.

"*Immediately after the tribulation of those days shall the sun be darkened, and the moon shall not give her light, and the stars shall fall from heaven, and the powers of the heavens shall be shaken. And then shall appear the sign of the* SON OF MAN *in heaven: and then shall all the tribes of the earth mourn, and they shall see the Son of Man coming in the clouds of heaven with power and great glory. And he shall send his angels with a great sound of a trumpet, and they shall gather together his elect from the four winds, from one end of heaven to the other.*"

Such was the solemnly grand symphony which struck upon the disordered chords of Haydon's morbid imagination, as he stood upon the brink of eternity—as he hovered between the PAST and the FUTURE—ready for the self-commanded plunge. The mighty stars falling, the darkened orbs of day and night, the crash of the shaking "powers," the mourning multitudes of all the tribes of the earth in vast illimitable perspective, the glorious vision of the Son of Man in radiant masses of cloud, and the angels, and the clanging trumpets—gigantic—hoarsely pealing the call from death to life of the sleeping myriads—all these would probably rush through the poor sufferer's imagination during that terrible ordeal, and perhaps arrested for a moment his suicidal hand.

The moral of Haydon's story lies upon the surface. It is none other than that drawn by his own pen. He says, when analyzing the character of his intellect, "*My mind wanted the discipline of early training. I trace all the misfortunes in my life to this early and irremediable want.*" What a lesson does this confession teach us! Early training—early mental discipline—self control—self denial—mastery over the passions—how much of our after happiness depends upon the steady cultivation of such habits of mind! Poor Haydon had early and fond aspirations for the study of art. When he mentioned the bias of his mind, and said that he had resolved to be a painter, his father appearing to have some gloomy forebodings as to the future, replied, "Then you will live to repent." Haydon promptly rejoined "Never, my dear father; *I would rather die first!*" Although only fifteen years of age when this conversation occurred, he seems to have had, even at that early period of his chequered life, a shadowy and prophetic conception of his unhappy destiny. In forty-eight years from the date of this conversation, his grey hairs were bedabbled with his own blood!

